# Stable HIV-1 envelope glycoprotein immune complexes as vaccine immunogens

**DOI:** 10.1186/1742-4690-8-S2-P75

**Published:** 2011-10-03

**Authors:** Thijs van Montfort, Mark Melchers, Tony MM van Capel, Ester C de Jong, William A Paxton, Rogier W Sanders

**Affiliations:** 1 Laboratory of Experimental Virology, Department of Medical Microbiology, Academic Medical Center of the University of Amsterdam, Amsterdam 1105AZ, The Netherlands; 2 Department of Cell Biology and Histology, Academic Medical Center, University of Amsterdam, Amsterdam 1105AZ, The Netherlands

## 

The development of an HIV-1 vaccine that elicits strong neutralizing antibody (nAb) and T cell responses is challenging. Classical vaccine strategies such as live attenuated vaccines are considered unsafe whereas envelope glycoprotein (Env)subunit vaccines induce low nAb titers that do not protect against HIV-1 infection. We showed previously that most HIV-1-antibody immune complexes (HIV-ICs) formed with either broadly nAbs or Abs derived from patient sera dissociate into free HIV-1 virions and Ab when captured by dendritic cells (DCs). Dissociation of HIV-ICs allows for transmission from DCs to CD4^+^ T target cells. H but more importantly it can hamper the activation of immune cells which is a hallmark of stable ICs. The natural role of ICs is enhancing uptake by DCs, DC activation, induction of antigen presentation and induction of T cell responses. Furthermore, ICs are captured by follicular DCs that activate the B cells for Ab production, Ab affinity maturation and ísotype switching. We explore stable Env-ICs as a vaccine candidate. To form stable Env-ICs we fused the Fc-region of immunoglobulins to trimeric gp140. Env-IC maintained a native Env conformation which was evaluated by ELISA with Env-specific Abs. Native PAGE analyses and size exclusion chromatography showed that Env-ICs formed trimers, but hexamers consisting of 2 Env trimers and 3 dimeric Fc-tails were also observed. The functionality of the Fc-tail was evaluated by immuno-precipitation of the Env-IC with protein-G couple beads. Capture of Env-IC by DCs was enhanced with 50% compared to wild-type Env. Moreover, Env-IC captured by DCs more efficiently activated gp120-specificT helper cells.

